# Multidimensional advice networks in primary health care

**DOI:** 10.1017/S1463423626101327

**Published:** 2026-06-16

**Authors:** Ilknur Aydin Teker, Fatma Mansur

**Affiliations:** 1 Independent Scholar, Türkiye; 2 Department of Healthcare Management Faculty of Economics and Administrative Sciences, Ankara Hacı Bayram Veli University, Ankara, Türkiye

**Keywords:** advice interaction, primary health care, social network, social network analysis

## Abstract

**Aim:**

We aimed to examine advice interactions among family physicians using social network analysis (SNA) by categorizing advice interaction according to the five advice dimensions.

**Background:**

Inter-individual interactions for information exchange is a powerful tool for the pursuit of solutions to issues. These interactions may involve advice-seeking.

**Methods:**

The whole network approach was adopted and face-to-face research was conducted with 139 family physicians. Data were analysed using social network software, UCINET and visualized using the NETDRAW software. To examine the multidimensional advice networks, the frequency, density, reciprocity (dyad) measures were used. The Quadratic Assignment Procedure was used in UCINET to measure the correlations between the dimensions of advice. The Girvan–Newman algorithm was used to examine clustering in the advice network.

**Findings:**

Density values in the advice dimensions were very low. This indicates that the network was sparse, with limited interactions among family physicians in terms of giving and receiving advice. The strength of the ties in the dimensions was realized through validation, solutions, problem reformulation, meta-information, and legitimization, respectively. The results showed that the relationships between the dimensions were moderately, positively and significantly correlated. The advice network exhibited high modularity. Family physicians tended to seek advice from colleagues at the family health centers where they worked. We presented a visual representation of advice networks in primary healthcare settings. Identifying multidimensional advice networks through social network analysis can provide insight into how information is disseminated among family physicians. Our findings could contribute to decision makers in developing solution-oriented processes.

## Introduction

A social network is a set of socially relevant nodes connected to each other by one or more relationships. These nodes are typically persons or organizations but can also be countries, neighbourhoods, departments within organizations, web pages, or magazine articles (Marin and Wellman, 2011). In sociological studies, nodes are often referred to as ‘actors’ (Otte and Rousseau, 2002). Health service researchers are increasingly interested in networks because many medical and health-related phenomena involve interdependent actors (e.g., patients, nurses, doctors, and hospitals) (O’Malley and Marsden, 2008).

Given the pivotal role of physicians in healthcare, it is crucial to comprehend the social networks within which they interact (Coleman *et al*., 1966). The exponential growth of the medical literature makes it difficult for physicians to stay current with the literature. Textbooks and journal articles are significant sources of information for many physicians, especially given the pervasive availability of electronic access to these sources (Haug, 1997; Bates and Gawande, 2003). Nevertheless, physicians frequently rely on their colleagues for new information to assist them in interpreting medical literature and obtaining specific advice regarding patient care. (Weinberg *et al*., 1981; Cullen, 1997; Keating *et al.*, 1998). Moreover, numerous physicians regard their colleagues as the most valuable sources of information (Gruppen *et al*., 1987; Williamson *et al*., 1989; Cullen, 1997). The knowledge and beliefs of colleagues and how they understand and share knowledge influence the manner in which doctors learn and the care they provide to their patients (Keating *et al*., 2007).

Interactions involving the transmission of information and other resources are common in the workplace. These interactions include seeking advice, co-operating, helping, supporting, or providing feedback (Agneessens and Labianca, 2022). Advice networks are relationships in which individuals share resources, such as information, help, and guidance, related to the completion of their work (Sparrowe *et al*., 2001). To determine what people get from others when they turn to them for information or advice, Cross (2000) conducted in-depth interviews with managers in a global consulting organization. He found that, in this context, people helped others in five unique ways. They tended to provide solutions, meta-knowledge, problem reformulation, validation, and legitimation. Another study examined these dimensions using systematic network analysis with a different sample. The transfer of each benefit has been evaluated as a social relationship. The search for information constitutes an advice relationship when a task performed by an individual improves with information obtained from others. Advice relationships can take many forms, including the solution to a problem, the means of obtaining information about solutions to that problem, a predefined solution, and the reliability of the proposed solution (Cross *et al*., 2001a).

The literature contains studies on information and advice-seeking in the health sector (Keating *et al*., 2007; Creswick and Westbrook, 2010; Bradley *et al*., 2011; Van Beek *et al*., 2011; Yuce *et al*., 2014; Creswick and Westbrook, 2015). A systematic analysis revealed that over half of the social network analysis (SNA) studies conducted in the health sector were conducted in secondary or tertiary care settings (Chambers *et al*., 2012). Research frequently provides insights into the interactions between individuals and organizations in the pursuit of solutions to specific issues. However, the evaluation of advice-seeking in businesses is often limited to a single dimension, potentially overlooking other crucial aspects of the advice network. Consequently, it is essential to extend the scope of analysis beyond advice networks to uncover the various dimensions of advice (Cross *et al*., 2001a).

As the introduction and application of SNA to public health systems and services research is relatively new in Türkiye, a network-based analysis may be appropriate for visualizing, describing, and analysing the primary healthcare system. Many SNA studies have only examined one type of advice network. However, since the structure of advice networks may vary depending on the type, analysing multiple advice links adds more dimensions to this research.

In Türkiye, primary healthcare services are provided through the family medicine system. Primary healthcare is a complex system characterized by dynamic patterns of interaction between members of the system and their environments (Miller *et al*., 2001; Crabtree, 2003). SNA is a valuable tool for quantitatively analysing complex systems represented by primary care practices. This tool has many potential applications, including its use to assist in the design of interventions to promote organizational change (Scott *et al*., 2005).

The exploration of various networks of information-seeking/advice interactions among family physicians using social network analysis can be critical for different health outcomes at different times and stages. Knowledge on the kinds of advisory networks doctors form to seek and share information can offer insights on how these networks benefit patients. New knowledge can be created among family physicians when much denser networks are formed through the sharing of information; this knowledge can be transferred and problems affecting patients can be better solved collectively.

Therefore, this study was conducted to examine advice interactions among family physicians in primary healthcare using SNA. The advisory network among family physicians was examined through systematic network analysis by considering each of the five types of benefit dimensions as a separate social relationship type.

The following research question was addressed:

1. What are the characteristics (in terms of the five dimensions in the advice interaction) of networks between family physicians in primary health care? Specifically:

- What is the density of advice interactions?
- How often is advice requested on average?
- Is there a strong advice link among family physicians?
- Are advice interactions reciprocal?

1. It can be posited that if there is a link between two actors in one dimension of advice, there is likely to be a link between them in another dimension of advice.

1. Is there a clustering in the network?

## Methods

### Setting and sample

We employed a spatial whole-network approach in which the network actors were family physicians registered in a district in Ankara, which is among the central districts but outside the metropolitan area. Although the district is farther from the centre than the other districts, it was chosen because it has a higher population density, which makes it home to a considerable number of family physicians and family health centres (FHC), as well as a high rate of family physician visits.

This district selection criterion is also based on a study that supports the argument that the personal networks of non-metropolitan residents tend to be more multiplexous and long-term than those of metropolitan residents (Beggs *et al.* 1996). Furthermore, the conditions in these settings facilitated the development of these networks. The formation of collaborative relationships is supported by previous studies (Bradley *et al*., 2011; Bradley *et al*., 2012).

This study was conducted face-to-face with 139 family physicians from 34 FHC in the designated district. Overall, 81% of the family physicians participated in the study. Table 1 presents the definitions of the terms ‘family health center’ and ‘family physician’, as established by the pertinent legislation.

**Table 1.** Abbreviations and explanations Table 1 long description.

Family Health Centre (FHC)	Primary health care facility where family medicine services are provided with one or more family physicians and other professionals (midwives/nurses).
Family physician	These may include family medicine specialists, or specialized physicians or general practitioners who have received training prescribed by the institution.

FHC, family health centre.

### Social network questionnaire

Data were obtained through a social network questionnaire.

The social network questionnaire employed in this study is a synthesis of insights derived from previous studies on social networks, information seeking, and advice interactions (Cross, 2000; Cross *et al*., 2001a, 2001b; Lazega, 2001; Sparrowe *et al*., 2001; Borgatti and Cross, 2003; Cross and Sproull, 2004; Scott *et al*., 2005; Creswick and Westbrook, 2010; Bradley *et al*., 2011; Erdogan *et al*., 2014).

The social network questionnaire used questions from Cross *et al*. (2001a) to ascertain the dimensions of the advice network: each of the five dimensions (solutions, meta-knowledge, problem reformulation, validation and legitimation) is operationalized as a specific type of social relationship. Family physicians were posed five distinct queries pertaining to advice rather than a single general question. They were instructed to provide the names of colleagues with whom they had engaged in advice-seeking interactions within the previous 6 months. Additionally, the frequency of contact was calculated based on the previous 6 months, rather than the previous month. The Cross *et al*. (2001a) study consists of a network analysis of senior executives who recently made a significant acquisition. This study does not seek advice relationships for a specific project. Given the diversity of patients visiting family physicians, a longer period is required to identify advice networks, and as noted in Cross’s (2000) study, the last six months were used as the reference period. Table 2 delineates the survey questions pertinent to the advice dimensions.

**Table 2.** Network questions^*^ Table 2 long description.

1. (Solutions) Indicate the extent to which you have turned to each of the following people within the last six months for answers to fairly specific or detailed questions at work.
2. (Meta-knowledge) Indicate the extent to which you have turned to each of the following people within the last six months for general guidance or advices to other sources of information.
3. (Problem reformulation) We often turn to other people for their ability to help us think through a problem even when they may not have specific information that we need. Such people help us consider various dimensions of a problem and anticipate issues and concerns likely to appear in the future. Indicate the extent to which you have turned to each of the following people within the last six months for such assistance.
4. (Validation) Often we turn to other people and do not receive any information whatsoever. However, being able to talk through ideas with another person bolsters self-confidence and thinking and makes you more willing to introduce your ideas to others and more confident in expressing them. Indicate the extent to which you have turned to each of the following people within the last six months for such a purpose.
5. (Legitimation) Sometimes we turn to someone for information and advice solely for the ability to say we have spoken with that person about our ideas. The individuals may be in a higher position within the organization or a perceived expert in a given area. Indicate the extent to which you have turned to each of the following individuals within the last six months for such a purpose.

* Cross *et al*., 2001a.

In this study, a name-generator approach, which involves asking participants to name individuals with whom they have a specific relationship, was employed (Agneessens and Labianca, 2022). The names of at least three individuals were requested with no upper limit specified. Detailed definitions of the questions were provided, elucidating the meaning of advice for each dimension and striving to ensure question standardization in accordance with the approach adopted by Brewer (2000).

### Construction of the SNA matrix

**Figure 1.**
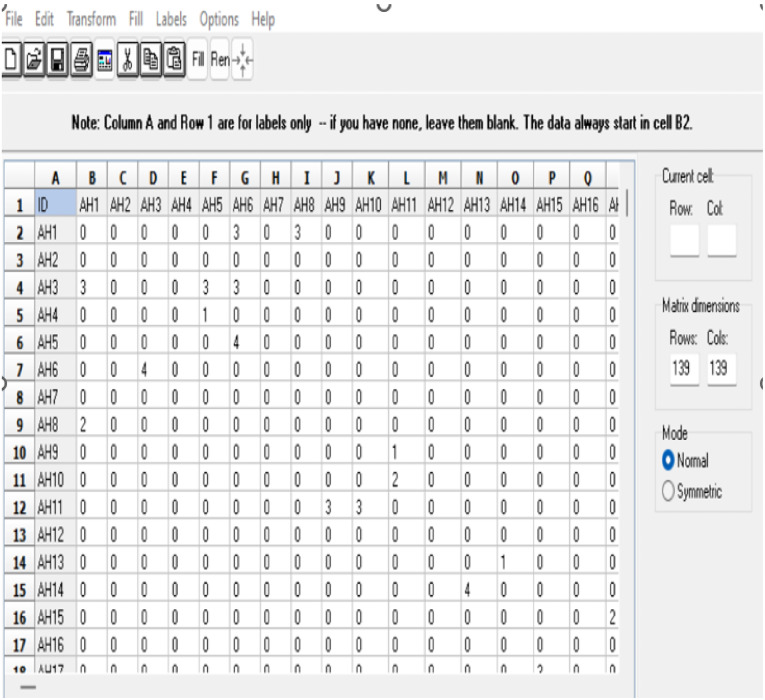
Figure 1 long description. Adjacency matrices. The presence of an advice interaction in any advice dimension between each pair of family physicians was indicated using response categories, ranging from 1 to 4, whereas its absence was indicated by the number ‘0’.

Data were analysed using the university of California, Irvine network (UCINET), a social networking software. The network analysed was unimodal because it contained only one type of node (family physicians). First, the survey data were entered into Excel spreadsheets and imported into UCINET. The data were organized using 139 × 139 square matrices, with all members of the network listed in both the rows and columns of the matrix (Figure 1). In the matrix, the presence of an advice interaction in any advice dimension between each pair of family physicians was indicated using response categories, ranging from 1 to 4, whereas its absence was indicated by the number ‘0’. Separate matrices were created for each advice relationship, and the data were organized and analysed separately. Relational data were visualized using network maps (sociograms) produced using NETDRAW within the UCINET program. The names provided by the participants in the social network questionnaire were kept strictly confidential in the study, and family physicians were coded as ‘AH1’, ‘AH2’ to ‘AH139’ in the analyses with a ranking technique determined by the researcher.

### SNA quantitative measures

The structural characteristics of each dimension in the advice network were analysed using SNA measures. We used the frequency of advice (frequency of advice-seeking in the previous 6 months, ranging from 1 to 4), density (the ratio of the number of ties to the number of potential ties in each dimension), and reciprocity (in which the advice relationship is reciprocal). The Quadratic Assignment Procedure in UCINET was used to measure the correlations between the advice dimensions. The Girvan–Newman algorithm was used to examine clustering in the advice network.

### Frequency of advice

The frequency of advice interactions among network members was measured in relation to each dimension. This measure is obtained by dividing the total value of all ties by the number of actual advice ties. The total value of all ties was calculated with reference to the response category (Table 3) as follows: under the heading ‘frequency/frequencies’ in the UCINET program, the distribution of advice ties in each dimension according to the answer categories was calculated. For example, in the solutions dimension, it was found that ‘4’ was given in 56 ties, ‘3’ in 26 ties, ‘2’ in 24 ties, and ‘1’ in 33 ties. These response categories were multiplied by the number of ties and calculated as (4x56) + (3x26) + (2x24) + (1x24) + (1x33) = 383. The total value of all ties was calculated. By dividing this value by the number of links realized in the relevant dimension, the frequency of advice interactions of family physicians in the previous 6 months was calculated based on the response category. This average value was interpreted using response categories ranging from 1 to 4, as (Table 4).

**Table 3.** Response scale Table 3 long description.

Response	Frequency of advice-seeking categories
0	Not once during the last six months.
1	1–2 times during the last six months
2	3–4 times during the last six months.
3	5–6 times during the last six months.
4	7 or more times during the last six months.

### Community detection

In a social network, a community can be defined as a group of people connected by a common interest, shared location or workplace, or family ties (Newman and Girvan, 2004; Newman, 2012). Communities are referred to as clusters or modules (Fortunato, 2010). The ability to identify and examine clusters is crucial for comprehending and visualizing the configuration of networks (Girvan and Newman, 2002; Newman and Girvan, 2004; Fortunato, 2010).

**Figure 2.**
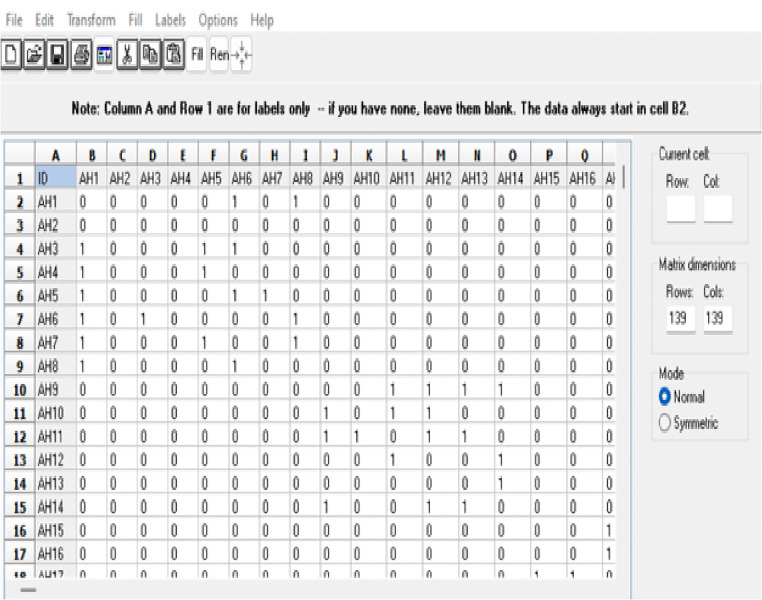
Figure 2 long description. Matrix for community analysis. The presence of an advice interaction in any advice dimension between each pair of family physicians is indicated by the number ‘1’, whereas its absence is indicated by the number ‘0’ in the matrix.

A community analysis was conducted to examine the community structure and identify the clusters present within the advice network. The Girvan–Newman algorithm (Girvan and Newman, 2002) was used for community analysis. A matrix was created for community analysis (Figure 2). The presence of an advice interaction in any advice dimension between each pair of family physicians is indicated by the number ‘1’, whereas its absence is indicated by the number ‘0’ in the matrix.

In the Girvan–Newman algorithm, the concept of modularity is used to quantify community structure strength. Modularity is a metric that verifies the most accurate partitioning of a network into clusters, communities, or modules (Newman and Girvan, 2004; Newman, 2012).

## Results

### Frequency of advice

The average frequency of advice according to the response category is the highest in the validation dimension and lowest in the legitimization dimension. The mean value for the frequency of advice interaction between family physicians in the validation dimension was 3.23, which shows that in this dimension, family physicians asked for advice five to six times on average in the previous 6 months (Table 4).

**Table 4.** Frequency of advice interaction Table 4 long description.

Dimensions of the advice network	Total value	number of ties	Response categories	Frequency of advice-seeking
Solutions	383	139	2.75	>3–4 times during the last six months
Meta-knowledge	363	144	2.52	>3–4 times during the last six months
Problem reformulation	359	136	2.63	>3–4 times during the last six months
Validation	689	213	3.23	<5–6 times during the last six months
Legitimization	312	134	2.32	<3–4 times during the last six months

The frequency of advice in the dimensions also indicates the average strength of the ties (Wasserman and Faust, 1994). The strength of the ties in the dimensions was ranked as follows: validation, solutions, problem reformulation, meta-knowledge, and legitimization.

### Density

The density values observed in the data ranged from 0 to 1 (Scott, 2000). Density values in the advice dimensions were notably low indicating a sparse network in each benefit dimension in terms of advice interaction (Table 5).

**Table 5.** SNA quantitative measures Table 5 long description.

Advice dimensions	Density	Reciprocity (dyad)
Solutions	0.007	0.23
Meta-knowledge	0.008	0.25
Problem reformulation	0.007	0.28
Validation	0.011	0.43
Legitimization	0.007	0.19

SNA, social network analysis.

### Reciprocity

The reciprocity values for the dimensions were not high. Values approaching 1, which represents the maximum value in practice, indicate a network structure with high reciprocity. In this research, to the dyad method, the highest reciprocity was observed in the validation dimension. Consequently, 43% of all actor pairs that had any advice connection in the validation had a reciprocal connection, implying that 43% of the family physicians who sought advice in the validation dimension were sought out for advice by the same individuals (Table 5).

**Figure 3.**
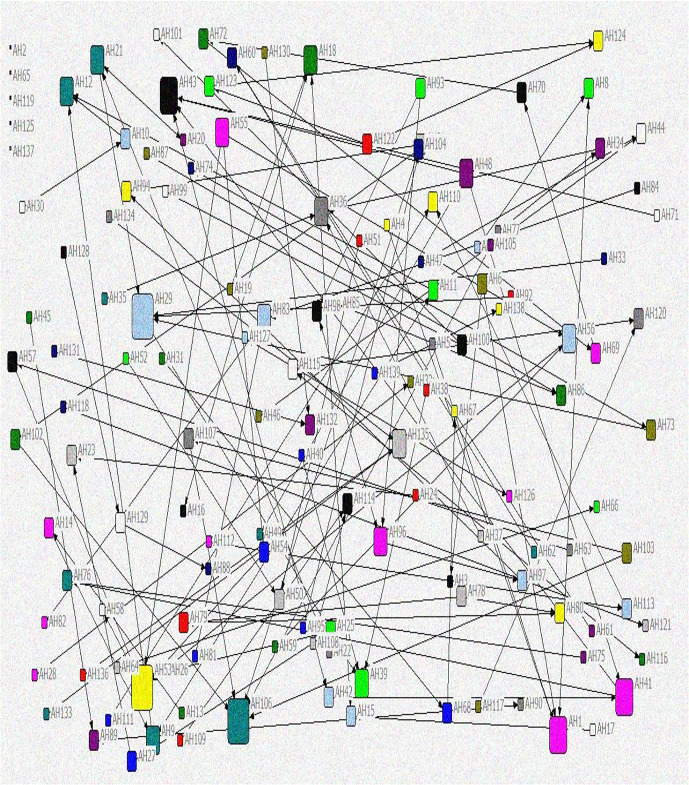
Figure 3 long description. Solutions. Each node (actor) is indicated by a rectangular sign of a different colour representing family physician. The size of the rectangle is directly proportional to the number of advice interactions with the relevant actor. The lines between the nodes in the network indicate the advice ties realized in the network. Bidirectional arrows in the network indicate reciprocity. The list on the left side of the figure indicates the isolated actors who did not have advice interactions in the relevant dimension.

**Figure 4.**
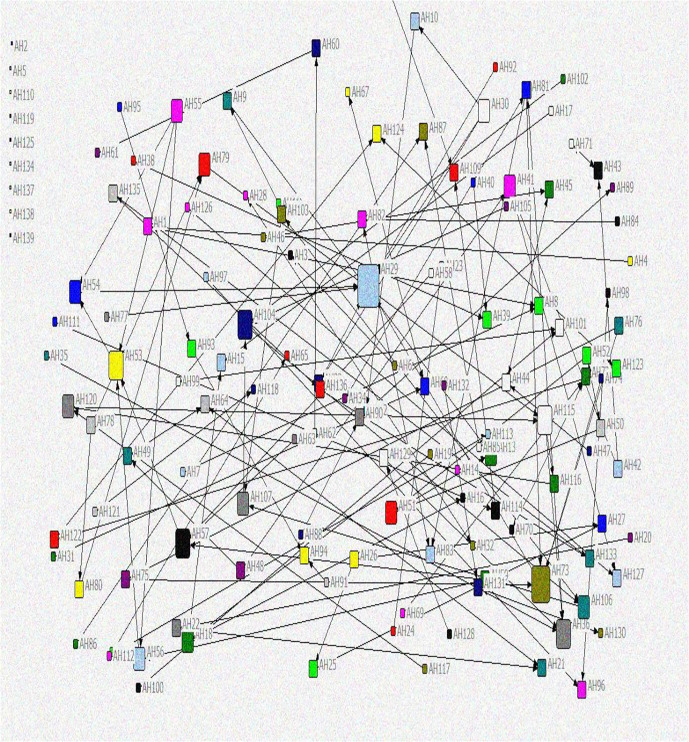
Figure 4 long description. Meta-knowledge. Each node (actor) is indicated by a rectangular sign of a different colour representing family physician. The size of the rectangle is directly proportional to the number of advice interactions with the relevant actor. The lines between the nodes in the network indicate the advice ties realized in the network. Bidirectional arrows in the network indicate reciprocity. The list on the left side of the figure indicates the isolated actors who did not have advice interactions in the relevant dimension.

**Figure 5.**
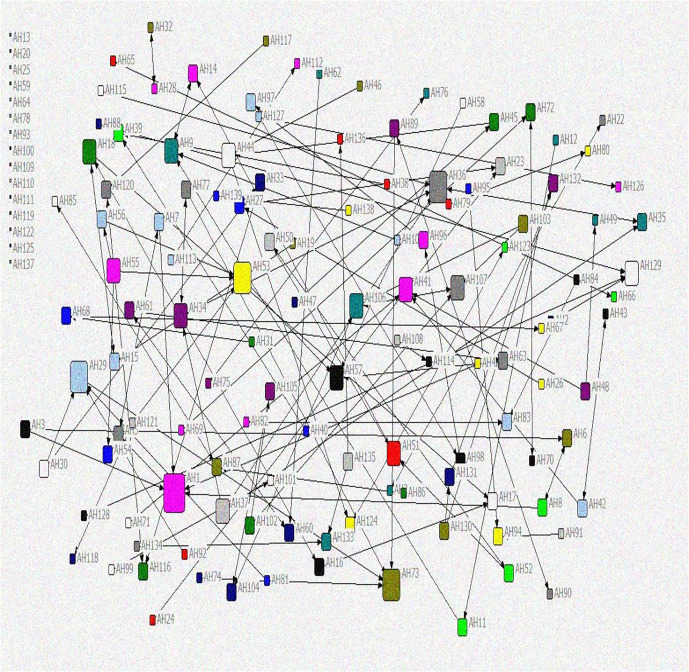
Figure 5 long description. Problem reformulation. Each node (actor) is indicated by a rectangular sign of a different colour representing family physician. The size of the rectangle is directly proportional to the number of advice interactions with the relevant actor. The lines between the nodes in the network indicate the advice ties realized in the network. Bidirectional arrows in the network indicate reciprocity. The list on the left side of the figure indicates the isolated actors who did not have advice interactions in the relevant dimension.

**Figure 6.**
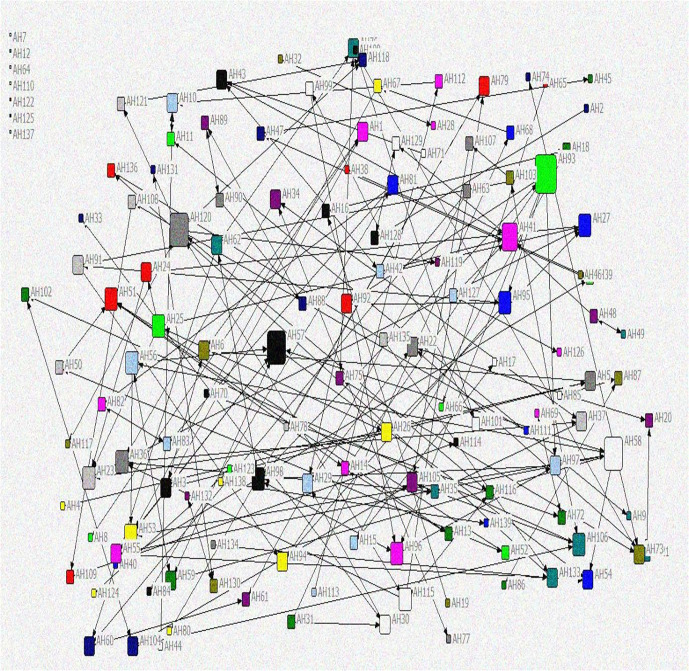
Figure 6 long description. Validation. Each node (actor) is indicated by a rectangular sign of a different colour representing family physician. The size of the rectangle is directly proportional to the number of advice interactions with the relevant actor. The lines between the nodes in the network indicate the advice ties realized in the network. Bidirectional arrows in the network indicate reciprocity. The list on the left side of the figure indicates the isolated actors who did not have advice interactions in the relevant dimension.

**Figure 7.**
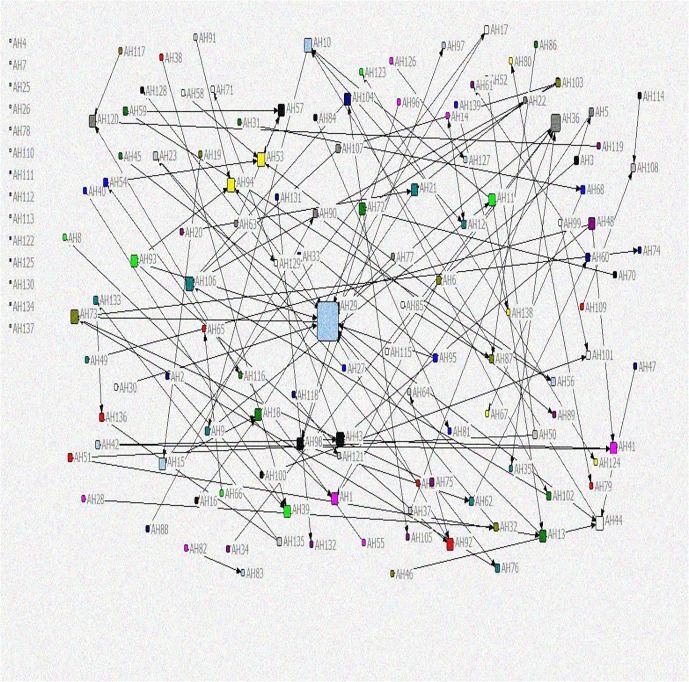
Figure 7 long description. Legitimation. Each node (actor) is indicated by a rectangular sign of a different colour representing family physician. The size of the rectangle is directly proportional to the number of advice interactions with the relevant actor. The lines between the nodes in the network indicate the advice ties realized in the network. Bidirectional arrows in the network indicate reciprocity. The list on the left side of the figure indicates the isolated actors who did not have advice interactions in the relevant dimension.

Sociograms for each dimension of the advice network are presented in Figures 3–7. The dimension of problem reformulation had the highest number of isolated family physicians (15 isolates).

### Relationship between the dimensions of advice

When one has knowledge of multiple relationships among the same set of actors, it is often of considerable interest to ascertain whether the probability of one type of link is related to that of another. For example, if actors exchange advice to solve a specific problem, can this create a sense of trust, making the advice relationship more likely in the reformulation of a problem? This is because pairs of actors who engage in one type of exchange are more likely to engage with one another. Alternatively, the two relationships may be independent and exhibit no correlation.

Table 6 illustrates the correlations between the advice dimensions. The correlation results demonstrate that if there is a link between two family physicians in one advice dimension, there is a probability that they will have a link in another dimension of advice. For example, one of the highest correlations is 0.68. This indicates that if there is 1 in a cell of the legitimation matrix, there is a 68% probability that there is 1 in the corresponding cell of the problem reformulation matrix. Consequently, there is a moderately positive and significant relationship between these two dimensions. The relationships between all the dimensions of the advice network were positive and significant. The relationships between the dimensions were moderately correlated (had a correlation coefficient value between 0.3 and 0.7, indicating a moderate level of relationship) (Gurbuz and Sahin, 2016).

**Table 6.** Correlation between the dimensions of advice* Table 6 long description.

	Solutions	Meta-knowledge	Problem reformulation	Validation	Legitimization
Solutions	1.00	–	–	–	–
Meta-knowledge	0.61	1.00	–	–	–
Problem reformulation	0.55	0.61	1.00	–	–
Validation	0.47	0.52	0.50	1.00	–
Legitimization	0.61	0.64	0.68	0.49	1.00

* All correlations were statistically significant at *p* < 0.01 level using the Quadratic Assignment Procedure permutation test.

One of the lowest matches had correlation coefficient 0.47, indicating a positive correlation between the two advice dimensions (validation and solution). Nevertheless, Hanneman and Riddle (2005) contended that although these correlation values appear to indicate a relationship because of the density of the two matrices, matching randomly rearranged matrices may also demonstrate an average match of 0.475, suggesting that the observed measurement is not significantly different from a random result.

### Community detection

**Figure 8.**
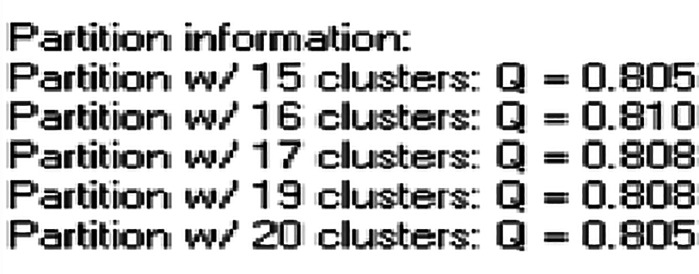
Figure 8 long description. Modularity values of the network.

The modularity coefficients of the network exhibited a decline when the number of clusters was either below or above 16 (Figure 8).

The general advice network exhibited the highest modularity (*Q* = 0.810) when partitioned into 16 clusters. Values approaching *Q* = 1, the maximum value, indicated networks with strong community structures. In practice, values are generally within the range of 0.3–0.7. Higher values are rare (Newman and Girvan, 2004). However, in this study, the network was divided into clusters with high modularity coefficients (0.8). Therefore, the advice network had high modularity.

**Figure 9.**
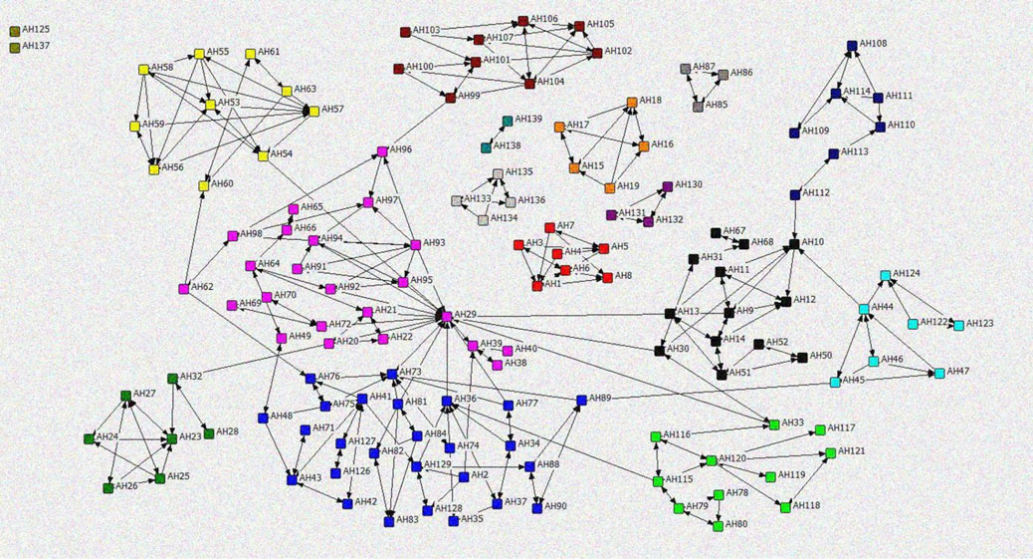
Figure 9 long description. Clusters in the network. The colours in the network represent the communities/clusters formed according to the modularity coefficient. Each node in the clusters is represented by a rectangle that represents a family physician. The lines between the nodes in the network indicate the advice ties realized in the network. Bidirectional arrows in the network indicate reciprocity. There are two isolated family physicians (AH 125 and AH137) in the upper left corner of the network. Isolates are considered as a separate cluster.

**Figure 10.**
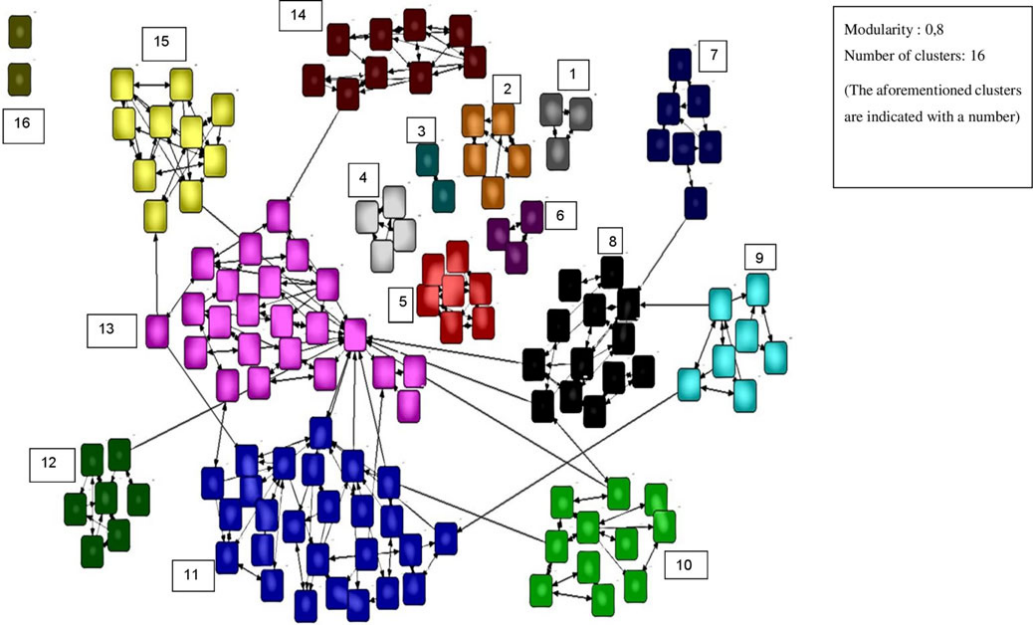
Figure 10 long description. Clusters in the network. The colours in the network represent the communities/clusters formed according to the modularity coefficient. Each node in the clusters is represented by a rectangle that represents a family physician. The lines between the nodes in the network indicate the advice ties realized in the network. Bidirectional arrows in the network indicate reciprocity. There are two isolated family physicians in the upper left corner of the network. Isolates are considered as a separate cluster. The clusters are numbered and illustrated in Figure 10.

The resulting sociograms of the clusters are shown in Figures 9 and 10. The colours in the network represent the communities/clusters formed according to the modularity coefficient. There are isolated family physicians in the upper left corner of the network. Isolates are considered as a separate cluster.

The network was divided into clusters characterized by dense intra-cluster connections and less dense inter-cluster connections, indicating dense intra-cluster advice links but fewer inter-cluster links (Figures 9 and 10).

Notably, the various clusters within the network exhibited disparate structural characteristics. Cluster 13 appeared to be organized around a more central area. AH29 occupied a central position within this cluster of advice networks. Clusters 7–12, 14, and 15 appeared to be loosely knit groups of collaborative nodes that could work together in various combinations, where no single actor is active, and different actors interact with each other, especially Cluster 11. The largest number of family physicians (26 actors) was observed in Cluster 11. Among the clusters connected to others by at least one link, 113 family physicians constituted a large component. The six components (Clusters 1–6) exhibited only advice links among themselves, with no external advice links for family physicians.

The communities/clusters that emerged in this study closely align with the concept of a common workplace, more specifically with FHC where family physicians are employed; for example, cluster 1–6 were composed of family physicians working in the same FHC. AH53–59 (Cluster 15), AH23–27 (Cluster 12) and AH108–114 (Cluster 7) worked in the same workplace. Analysis of the sociograms revealed that advice connections among family physicians working at the same FHC were particularly strong. Consequently, family physicians tend to receive advice from colleagues at the same FHC. In the clusters between Clusters 7 and 15, it is evident that advice connections between some family physicians are still robust; however, there are also limited connections with family physicians in other FHCs.

## Discussion

In this study, we revealed that the densities of the advice dimensions were low. This indicates that in terms of giving and receiving advice, the network was sparse, with minimal interaction between family physicians.

Possible reasons for this low density in the advice network could include professional experience, preferring to consult specialists with more specialized knowledge rather than family physician colleagues who tend to be less specialized, and the integration of technological advancements, such as artificial intelligence (AI), into medical application areas.

A study found that primary care physicians acted rationally according to their knowledge, that is, the less a primary care physician knew, the more likely they were to prefer the involvement of a specialist in patient care and management in all scenarios. Primary care physicians with less knowledge were more likely to prefer assistance from neurologists (Swarztrauber *et al*., 2002)

Another study revealed that in primary care practice, doctors receive information from colleagues with greater expertise and experience as well as from colleagues who are conveniently accessible by location and schedule. This result shows that it may be possible to organize practices to encourage faster dissemination of high-quality evidence-based medicine (Keating *et al*., 2007).

With the integration of technological developments such as AI into medical practice, there may be less frequent advice interactions between physicians. In a systematic review aimed at determining the scope of the application of AI tools in decision-making processes within healthcare delivery networks, it was shown that AI tools are used at various stages of the healthcare decision-making processes. The use of AI can improve the quality, efficiency, and effectiveness of healthcare by providing accurate, timely, and personalized information to support decision-making (Khosravi *et al*., 2024).

The more actors are related to each other, the denser the network (Palonen and Hakkarainen, 2014). It has been demonstrated that a higher density of decision-making networks in primary healthcare practice indicates greater interaction among the individuals involved in the decision-making process (Scott *et al*., 2005). A study investigating the communication and advice networks of nurses working in long-term care found that the networks were relatively dense, consistent with the high level of cooperation required to provide optimal care to patients. Furthermore, the results indicated that communication networks are crucial for staff job satisfaction (Van Beek *et al*., 2011).

It is also notable that reciprocity values in the advice dimension were relatively low. In general, they were below 50%. Given that the intensity values in the dimensions were very low, it was not surprising that the reciprocity values were also low. Nonreciprocal ties indicate a lack of two-way information sharing and cooperation. Family physicians with high reciprocity in the advice dimension mostly worked at the same FHC. The reciprocity of ties is a significant feature of a social structure. The extent to which a network is characterized by reciprocal ties can provide information about the degree of cohesion, trust, and social capital (Hanneman and Riddle, 2005).

Family physicians sought advice three to four times, on average, in the previous 6 months across most of the dimensions. The dimensions in which advice interaction occurs more frequently show the knowledge gaps or problems that family physicians encounter in practice. Family physicians wish to obtain information from colleagues to find solutions to problems because answering questions ensures effective and timely management of the problem. There is also an interaction with regards to verifying the solution or plan. For instance, family physicians may seek advice because of knowledge gaps between family medicine legislation and practice, or a lack of knowledge regarding legal regulations. Social and technical interventions would be required to facilitate information flow in advisory networks. In areas where the search for solutions and information is intense, the issuance of explanatory documents on the implementation of guidelines and directives by health authorities may be helpful. It is thought that more intensive networks can be built by sharing these explanatory documents, which will enable the generation of new knowledge among family physicians, the validation and dissemination of this knowledge, and thus, the collective resolution of problems. This situation provides opportunities for decision-makers. Leveraging these capabilities on the network is also important for the public image.

The frequency of advice interactions in the dimensions is also important for demonstrating the average strength of the ties. The strengths of the ties in the dimensions were ranked as validation, solutions, problem reformulation, meta-knowledge, and legitimization, respectively. In a study conducted on managers with the same questions, the order was found to be solutions, meta-knowledge, problem reformulation, validation, and legitimization (Cross *et al*., 2001a).

The network perspective emphasizes the structure of relationships rather than the attributes of individual actors. This perspective allows for the identification of opportunities and constraints that influence behaviour and outcomes (Ackland and Zhu, 2015). Granovetter emphasizes the importance of weak ties as potential sources of innovative and useful information. He argues that weak ties are required for individuals to seize opportunities and integrate them into their communities. In contrast to strong ties, weak ties may result in individuals becoming disconnected from the rest of the network. However, this lack of connection may facilitate the acquisition of new sources of knowledge within the network, suggesting that weak ties are a crucial means by which individuals can identify and seize opportunities and integrate them into communities (Granovetter, 1973).

From this perspective, the findings present opportunities for family physicians to transfer resources and information. We postulate that advice interactions among family physicians in search of solutions or information can be powerful tools for the dissemination of information. By strengthening the advice network through communication strategies, it may be possible to introduce and disseminate new information into the network. It is necessary to implement various communication strategies to enhance the connectedness of advice networks and facilitate the dissemination of new information. Particularly, it is crucial to promote communication between physicians through the dissemination of health events such as in-service trainings or meetings. Additionally, encouraging and supporting the participation of family physicians in conferences or congresses can reinforce communication between family physicians and contribute to the exchange of ideas on both medical and non-medical issues. These activities should be organized at the national or international level by health authorities, such as the Ministry of Health, Provincial Health Directorate or professional organizations.

The correlation results indicated that if a bond exists between two family physicians in one advice dimension, there is a high probability that they will also have a bond in another type of advice. This can be because the bond formed between them through one type of advice facilitates communication by creating a sense of trust. If communication campaigns are employed, they may contribute to the recognition of family physicians as important information channels, increase awareness, and facilitate the formation of new advice interactions in different dimensions with different exchanges of ideas. The network density increases as the amount of advice given and received in the network, that is the number of ties, increases. The subsequent process facilitates the engagement of isolated family physicians in the network, enabling them to participate in advice interactions and access new sources of information. Zappa (2011) posited that interactions between doctors can be a powerful tool for the diffusion of innovation; however, it should be supported by other strategies, particularly communication campaigns.

Analysing a network by breaking it down into clusters/modules reveals the structural characteristics of the entire network. The cluster structure of this study indicates that family physicians tended to seek advice from their colleagues in the FHC where they were employed. Additionally, they engaged in advice interactions with colleagues in other FHCs that were accessible according to the advice dimension. The communities/clusters that emerged in this study closely align with the concept of a common workplace, more specifically with FHCs where family physicians are employed. This result is not surprising because, in a social network, a community is considered a group of people connected by a common interest, shared location, or workplace (Newman, 2012). Consequently, it can be postulated that the foundation of advice relations is not predicated by differing titles or statuses but rather by a relational variable shaped by the shared workplace environment. The study conducted by Tagliaventi and Mattarelli (2006) supports the idea that working side by side and having common organizational values are important foundations for knowledge transfer between professional groups belonging to different practice networks.

The advice network in this study has high modularity. High modularity indicates dense connections inside the module and sparse connections outside the module. The degree of modularity indicates the flexibility and maturity of an organization and its range of functions (Langlois, 2002). Simon (1962) argues that it is natural for human-made organizations (e.g., government institutions and enterprises) to exhibit high degrees of modularity. This is because the human brain processes information and makes modular decisions (Meunier *et al*., 2009). It is believed that modular organization provide flexibility because modules can adapt, mature, or disappear without significantly disrupting the entire system (Shao and Zavala, 2020).

The existence of social networks means that people and events are interconnected, thereby enabling health and healthcare to transcend the individual in ways that warrant attention from patients, doctors, policymakers, and researchers alike (Christakis, 2004). The application of SNA in health services research can yield new insights into the phenomena under investigation while contributing to the ongoing evolution of the social network methodology (O’Malley and Marsden, 2008).

This research methodology can be employed to investigate multidirectional advice networks among different family physicians in different regions. This also permits cross-regional comparisons, thereby enabling the examination of different network structure models. This is supported by qualitative studies that identify more detailed problems and contribute to the development of solutions.

Research in the field of SNA in primary healthcare has the potential to increase the current understanding of interactions among family physicians. The discovery of different social networks may contribute to the coordination of physicians’ activities within and between organizations. Therefore, future research should focus on social support or trust networks among family physicians, or cooperation networks between family physicians and the Ministry of Health or non-governmental organizations.

## Conclusion

Social networks represent a crucial variable that influences the outcomes of all processes, yet they remain invisible or inferable only through specific analyses.

We propose that mapping a multidimensional advice network can provide interventions that managers can use to improve a network’s ability to create and share knowledge. This approach presents decision-makers with several potential avenues for action.

## Strengths and limitations

One of the strengths of this study is that it was conducted face-to-face in a natural environment in which family physicians worked, thus allowing for the collection of maximum information. However, the study was conducted in a single district, limiting the generalizability of the results to the country.

In this study, family physicians were asked to recall individuals with whom they had had advisory interactions in the previous 6 months; therefore, considering that people with whom relationships are shared might be forgotten, there is a possibility of recall bias. However, to curb this bias, they were provided a more detailed description of the questions, which were used as retrieval cues.

## Data Availability

The data supporting the findings of this study are available upon request from the corresponding author. The data were not publicly available because of privacy concerns of the research participants.

## Table 1. Long description

A table with two rows and two columns defines the terms family health center and family physician. The first row explains that a family health center is a primary health care facility where family medicine services are provided by one or more family physicians and other professionals such as midwives or nurses. The second row defines a family physician as a specialist in family medicine or a general practitioner who has received training prescribed by the institution.

Navigate back to Table 1..

## Table 2. Long description

The table presents five dimensions of advice networks: solutions, meta-knowledge, problem reformulation, validation, and legitimation. Each dimension is associated with specific questions. The solutions dimension asks about turning to people for answers to specific questions. The meta-knowledge dimension inquires about seeking general guidance or advice. The problem reformulation dimension focuses on turning to people to think through problems. The validation dimension involves talking through ideas to bolster self-confidence. The legitimation dimension pertains to seeking validation for ideas from higher-positioned individuals. The table has five rows and one column, each row detailing a different dimension and its corresponding question.

Navigate back to Table 2..

## Figure 1. Long description

The matrix displays advice interactions between family physicians across various dimensions. Rows and columns are labeled with identifiers such as AH1, AH2, and so on, indicating different physicians. The matrix consists of 139 rows and 139 columns. Each cell contains a numerical value ranging from 0 to 4, where 0 indicates the absence of an advice interaction, and values 1 to 4 indicate the presence of such interactions. Notable patterns include clusters of higher values, suggesting frequent advice interactions among certain groups of physicians. The matrix is symmetrical, with the diagonal elements all being 0, indicating no self-interactions. This structure helps visualize the network of advice relationships among the physicians.

Navigate back to Figure 1..

## Table 3. Long description

The table presents a response scale for the frequency of advice-seeking interactions among network members over the last six months. It consists of five rows and two columns. The first column lists response values from 0 to 4, and the second column describes the corresponding frequency categories. Response 0 indicates no advice-seeking in the last six months. Response 1 indicates 1-2 times during the last six months. Response 2 indicates 3-4 times during the last six months. Response 3 indicates 5-6 times during the last six months. Response 4 indicates 7 or more times during the last six months.

Navigate back to Table 3..

## Figure 2. Long description

The matrix displays advice interactions between family physicians, with rows and columns labeled from AH1 to AH16. It consists of 139 rows and 139 columns, indicating the presence or absence of advice interactions in various dimensions. A ‘1’ signifies the presence of an advice interaction, while a ‘0’ indicates its absence. The matrix is symmetrical, with diagonal elements all being ‘0’. Notable patterns include clusters of ‘1’s, suggesting frequent advice interactions among specific groups of physicians.

Navigate back to Figure 2..

## Table 4. Long description

The table presents data on the dimensions of an advice network, focusing on total value, number of ties, response categories, and frequency of advice-seeking. It includes five rows and five columns. The columns are labeled Dimensions of the advice network, Total value, Number of ties, Response categories, and Frequency of advice-seeking. The rows are labeled Solutions, Meta-knowledge, Problem reformulation, Validation, and Legitimization. Solutions have a total value of 383, 139 ties, a response category of 2.75, and a frequency of advice-seeking of more than 3-4 times during the last six months. Meta-knowledge has a total value of 363, 144 ties, a response category of 2.52, and a frequency of advice-seeking of more than 3-4 times during the last six months. Problem reformulation has a total value of 359, 136 ties, a response category of 2.63, and a frequency of advice-seeking of more than 3-4 times during the last six months. Validation has a total value of 689, 213 ties, a response category of 3.23, and a frequency of advice-seeking of less than 5-6 times during the last six months. Legitimization has a total value of 312, 134 ties, a response category of 2.32, and a frequency of advice-seeking of less than 3-4 times during the last six months.

Navigate back to Table 4..

## Table 5. Long description

A table with five rows and three columns compares different advice dimensions with their corresponding density and reciprocity values. The columns are labeled ‘Advice dimensions’, ‘Density’, and ‘Reciprocity (dyad)’. The rows list ‘Solutions’, ‘Meta-knowledge’, ‘Problem reformulation’, ‘Validation’, and ‘Legitimization’. Density values range from 0.007 to 0.011, indicating sparse network interactions. Reciprocity values range from 0.19 to 0.43, showing varying levels of mutual advice exchange.

Navigate back to Table 5..

## Figure 3. Long description

A network diagram illustrates family physicians as nodes represented by rectangular signs of different colors. The size of each rectangle corresponds to the number of advice interactions with the relevant actor. Lines between nodes indicate advice ties, with bidirectional arrows signifying reciprocity. The list on the left side of the figure identifies isolated actors who did not engage in advice interactions. The diagram visually maps the interconnectedness and communication patterns among family physicians.

Navigate back to Figure 3..

## Figure 4. Long description

A network diagram illustrating advice interactions among family physicians. Each node is represented by a rectangular sign of varying colors, with the size of the rectangle proportional to the number of advice interactions involving that actor. Lines between nodes indicate advice ties, with bidirectional arrows signifying reciprocity. The list on the left side of the figure identifies isolated actors who did not engage in advice interactions within this dimension.

Navigate back to Figure 4..

## Figure 5. Long description

A network diagram illustrates family physicians as nodes represented by rectangular signs of different colors. The size of each rectangle corresponds to the number of advice interactions with the relevant actor. Lines between nodes indicate advice ties, with bidirectional arrows signifying reciprocity. The list on the left side of the figure identifies isolated actors who did not engage in advice interactions within the relevant dimension.

Navigate back to Figure 5..

## Figure 6. Long description

A network diagram illustrating advice interactions among family physicians. Each node is represented by a rectangular sign of varying colors, with the size of the rectangle proportional to the number of advice interactions. Lines between nodes indicate advice ties, and bidirectional arrows signify reciprocity. The list on the left side identifies isolated actors who did not engage in advice interactions.

Navigate back to Figure 6..

## Figure 7. Long description

A network diagram illustrates advice interactions among family physicians. Each node, represented by a rectangular sign of varying colors, corresponds to a different family physician. The size of each rectangle is proportional to the number of advice interactions involving that physician. Lines between nodes indicate advice ties, with bidirectional arrows signifying reciprocity. The list on the left side of the figure identifies isolated actors who did not engage in advice interactions within this dimension. The diagram highlights the interconnectedness and collaborative relationships among the physicians, reflecting the multiplex and long-term nature of their networks.

Navigate back to Figure 7..

## Table 6. Long description

The table presents correlation values between different dimensions of advice, including Solutions, Meta-knowledge, Problem reformulation, Validation, and Legitimization. Each row and column header represents one of these dimensions. The correlation values range from 0.47 to 1.00, indicating varying degrees of relationship between the dimensions. For instance, the correlation between Solutions and Meta-knowledge is 0.61, while the correlation between Problem reformulation and Legitimization is 0.68. The table highlights that there are positive and significant relationships between all dimensions of the advice network, with correlation coefficients suggesting moderate levels of relationship.

Navigate back to Table 6..

## Figure 8. Long description

A table presents modularity values for partitions with varying numbers of clusters. The table has five rows and two columns. The first column lists the number of clusters, ranging from 15 to 20. The second column displays the corresponding modularity values. Notable values include 0.805 for 15 clusters, 0.810 for 16 clusters, 0.808 for 17 clusters, 0.808 for 19 clusters, and 0.805 for 20 clusters. The highest modularity value is 0.810, observed with 16 clusters.

Navigate back to Figure 8..

## Figure 9. Long description

The network diagram illustrates clusters of family physicians, represented by rectangles of different colors indicating communities or clusters formed based on the modularity coefficient. Lines between the nodes depict advice ties, with bidirectional arrows indicating reciprocity. Two isolated family physicians, labeled AH 125 and AH 137, are located in the upper left corner of the network. These isolates are considered separate clusters. The diagram highlights the interconnectedness and advice-seeking behavior among family physicians.

Navigate back to Figure 9..

## Figure 10. Long description

A network diagram displays clusters of family physicians, each represented by a rectangle. The colors indicate different communities or clusters formed based on the modularity coefficient. Lines between the nodes represent advice ties, with bidirectional arrows indicating reciprocity. Two isolated family physicians are shown in the upper left corner, considered separate clusters. The clusters are numbered and illustrated in the diagram.

Navigate back to Figure 10..
